# Improving outcomes of type 2 diabetes mellitus patients in primary care with Chronic Care Model: A narrative review

**DOI:** 10.1002/jgf2.659

**Published:** 2024-05-20

**Authors:** Arwa Ahmed Al‐Qahtani

**Affiliations:** ^1^ Department of Family Medicine, College of Medicine Al‐Imam Mohammed Ibn Saud Islamic University Riyadh Saudi Arabia

**Keywords:** Chronic Care Model, chronic disease, clinical outcomes, primary care, type 2 diabetes mellitus

## Abstract

Designed and implemented over two decades ago, the Chronic Care Model is a well‐established chronic disease management framework that has steered several healthcare systems in successfully improving the clinical outcomes of patients with type 2 diabetes mellitus. Research evidence cements the role of the Chronic Care Model (with its six key elements of organization of healthcare delivery system, self‐management support, decision support, delivery system design, clinical information systems, and community resources and policies) as an integrated framework to revamp the type 2 diabetes mellitus‐related clinical practice and care that betters the patient care and clinical outcomes. The current review is an evidence‐lit summary of importance of use of Chronic Care Model in primary care and their impact on clinical outcomes for patients afflicted with one of the most debilitating metabolic diseases, type 2 diabetes mellitus.

## 1 BACKGROUND

Among metabolic disorders, diabetes mellitus is the most frequent diagnosis globally. In the last 2 decades, a worrying rise in prevalence of diabetes mellitus among adults has been witnessed. In the year 2021, about 537 million individuals were living with diabetes mellitus. In the same year, diabetes mellitus was responsible for 6.7 million deaths. Every 1 in 2 individuals with diabetes mellitus go undetected. Economically, diabetes mellitus led to loss of approximately 966 billion dollars in global healthcare expenses in 2021. At present, about 541 million individuals are at high risk of developing type 2 diabetes mellitus.^1^

Urbanization and rampant socioeconomic advancement have significantly increased the influx of diabetes mellitus globally.^2^ Diabetes mellitus has considerable adverse impact on functional capacity and quality of life of affected patients, causing substantial morbidity and early mortality.^3^ Unhealthy eating habits, physically inactive lifestyle, alcohol consumption, and smoking, causing high body mass index, dyslipidemia, and elevated fasting plasma glucose are some of the risk factors attributed to severely increasing trends diabetes mellitus.^4^, ^5^ Old age is another risk factor that contributes to increasing incidence.^6^ Research data suggests that glycemic control continues to be less than the standard in patients with diabetes mellitus.^7^

Type 2 diabetes mellitus is the most common type that accounts for an approximate 90% of the cases.^8^ Therapeutic management of type 2 diabetes mellitus is a global health issue owing to snowballing number of new cases, healthcare costs, and complex care needs of these patients. As a result, physicians are often confronted to identify appropriate and efficient method of managing diabetes mellitus. The Chronic Care Model, stemmed through a comprehensive research in the United States by Wagner, offers an outline to effectively manage chronic diseases like diabetes mellitus by transforming key healthcare system components to facilitate top‐notch patient‐focused disease management.^9^, ^10^, ^11^

The Chronic Care Model has six key facets that are posited to influence clinical and functional outcomes related to the management of the disease. These six facets are (i) *Organization of healthcare delivery system*—refers to the development of an organizational ecosystem that provides and promotes highest quality health care and pledge to implement the Chronic Care Model across the healthcare organization; (ii) *Self‐management support*—refers to enabling, facilitating and supporting patients with the disease to become self‐sufficient to manage their health and well‐being; (iii) *Decision support*—refers to supporting patient care that liaise with emerging and up‐to‐date scientific evidence, healthcare guidelines, and patients' choices; (iv) *Delivery system design*—refers to coordination of healthcare delivery process, that is, ensuring effective, competent care, and facilitation self‐management support; (v) *Clinical information systems*—refer to ongoing monitoring of data and clinical outcomes in such a way that enables healthcare providers in making decision regarding efficient care delivery; and (vi) *Community resources and policies*—refers to development and utilization of community‐related resources and public health guidelines to encourage healthy regimes and sustain health and well‐being.^11^, ^12^

Healthcare organizations that implement and based on Chronic Care Model accomplish better clinical outcomes by and large. Therefore, integrating the Chronic Care Model in all stages of healthcare services must be emphasized.^13^, ^14^ A study by Wan et al.^15^ investigated the 5‐year effectiveness of Chronic Care Model in primary care setting in 53,436 patients diagnosed with type 2 diabetes mellitus. The study findings demonstrated that Chronic Care Model substantially decreased the new cases of clinical complications associated with type 2 diabetes mellitus and all‐cause death. Moreover, patients registered for the Chronic Care Model had their risk of cardiovascular disease reduced by 56.6%, microvasculature complications by 11.9%, and death by 66.1%. They also confirmed that the use of healthcare services reduced significantly in cohort with Chronic Care Model, which as a result caused lessening of healthcare expenditure by $7294 per person for the duration of the study.^16^

Ideal management of diabetes mellitus necessitates a systematized plan and the coordination of a team of healthcare personnel, involved in provision of a diabetes mellitus care, in an ecosystem that prioritizes patient and optimal care.^17^, ^18^ Chronic Care Model fits well with this strategy. Herein, the present review article aims to shed light on the importance of Chronic Care Model and their impact on clinical outcomes for patients afflicted with type 2 diabetes mellitus, in the light of recent research evidences, by discussing the current situation of type 2 mellitus diabetes mellitus globally; in the Gulf Cooperation Council countries and the Kingdom of Saudi Arabia; multifaceted and complex care needs of patients with chronic diseases; the role of primary care in effectively managing type 2 diabetes mellitus; the role of integrated care models in effectively reducing healthcare costs and hospitalizations; the notion Chronic Care Model; the key components of Chronic Care Model; scientific evidence of effectiveness of Chronic Care Model for adults with type 2 diabetes in primary care on improving patient outcomes; embracing Chronic Care Model for the management of patients with type 2 diabetes mellitus.

## 2 WHAT IS THE EXISTING SITUATION OF TYPE 2 DIABETES MELLITUS GLOBALLY?

Globally, about 462 million people are afflicted by type 2 diabetes mellitus, equivalent to 6.28% of the global population. In the year 2017, type 2 diabetes mellitus claimed over 1 million lives, ranked at 9th spot as the leading source of mortality from chronic diseases. This is a drastic increase in comparison with the year 1990, when type 2 diabetes mellitus was the 18th prominent cause of mortality. Both type 1 and type 2 diabetes mellitus included, diabetes mellitus is ranked as the 7th seventh leading cause of disability‐adjusted life years (DALYs), a measure of years of healthy life lost due to disease.^19^

According to Global Burden of Disease (GBD) data 1990 to 2017, managed by Institute of Health Metrics, the mounting prevalence of type 2 diabetes mellitus correlates with sociodemographic and economic development. For instance, European and American region demonstrate noticeably greater prevalence (8529 and 7060 per 100,000 population, respectively) and DALYs (842 and 1036 per 100,000 population, respectively) rates regardless of stringent public health actions.^19^

China, India, and the United States continue to be the major contributor to global pool of type diabetes mellitus patients due to their high population density, with 88.5, 65.9, and 28.9 million, respectively, affected by this condition. The prevalence is comparatively higher in males than females (6219 vs. 5898 per 100,000 population). It has further been reported that males are predisposed to early onset of type diabetes mellitus diagnosis, with prevalence being directly proportional to advancing age. The surge in worldwide diabetes mellitus prevalence, on the basis of GBD 1990–2017 data, is projected to be 7079 by 2030 and 7862 per 100,000 population by 2040.^19^ According to the International Diabetes Federation (IDF), the global prevalence of type 2 diabetes mellitus will be 783.2 million (12.2%) by 2045.^20^

## 3 HOW TYPE 2 DIABETES MELLITUS HAS AFFECTED GULF COOPERATION COUNCIL AND THE KINGDOM OF SAUDI ARABIA?

In countries of Gulf Cooperation Council, the documented prevalence of type 2 diabetes mellitus is between 4.3% and 34.9%.^21^ Between 2002 and 2018, high prevalence of type 2 diabetes mellitus among female population has been reported for Gulf Cooperation Council countries, with Qatar 10.8%, followed by United Arab Emirates 8%, Saudi Arabia 8%, Oman 8%, and Kuwait 5.4%.^22^ As per the 2019 report of IDF, Kuwait has the highest number of diabetes mellitus cases among six Gulf Cooperation Council countries. Yet, the large number of mortalities secondary to diabetes mellitus are documented from Saudi Arabia.^23^

In Saudi Arabia, the prevalence of type 2 diabetes mellitus is increasing owing to transition to high socioeconomic standards that have resulted in inactive lifestyle and poor dietary choices.^24^ It has been estimated that 13% of the Saudi population are living with type 2 diabetes mellitus in comparison with global prevalence of 10.5%.^20^ Furthermore, every 1 in 10 Saudi individuals are at increased risk of developing diabetes mellitus (prediabetes, a condition where there is hyperglycemia, but not extremely high to be established as type 2 diabetes mellitus).^25^ The modeling data suggest that the prevalence of type 2 diabetes mellitus raised from 8.5% in the year 1992 to 39.5% in 2022 in the Kingdom of Saudi Arabia.^26^ The increasing number of type 2 diabetes cases is also linked with growing prevalence of cardiovascular complications and early death.^27^ Nearly half (42%) of the deaths in the Saudi Arabia are reportedly caused by cardiovascular complications. It is estimated that the average cost of healthcare services for Saudi Arabian population with type 2 diabetes mellitus is 10 times higher compared with that of nondiabetic individuals.^28^

## 4 HOW INTEGRATED CARE MODELS HAVE PROVEN TO BE EFFECTIVE IN IMPROVING HEALTH OUTCOMES IN PRIMARY CARE?

Every so often, patients with chronic diseases are faced with disjointed care and therefore have to approach various health and social care facilities.^29^ The primary reason for this fragmented patient care is the incoherence between healthcare providers and dearth of individualized care and poor communication with patients.^30^ Therefore, effectively catering the needs of patients with chronic diseases is a mammoth task. In this regard, integrated care models have been shown to improve coordination among and within healthcare arena to better patient experience, satisfaction, and clinical outcomes.^31^, ^32^, ^33^

Integration can be described and utilized in numerous healthcare contexts, for instance, characterizing healthcare interventions that improves quality of care; however, without necessarily need of changing the way healthcare personnel carry out their tasks.^34^ Moreover, integrated care models can take diverse shapes, and a handful of interventions have been suggested. For example, the intervention of self‐management focuses on enhancing the patients' knowledge and attitude toward their own health and disease condition, promoting self‐care and medication compliance and adherence.^33^, ^35^

Likewise, other interventions like multidisciplinary teams, discharge management, and case management congregate to facilitate in developing effective communication among healthcare providers themselves and with patients across several healthcare setups.^36^ Apart from that, these interventions enhance care all along the continuum by effectively utilizing available resources, and thus helping in coordinating health and social care according to personalized needs of the patient with a sole objective to progress the quality of care and tackle fragmented care through continuing partnerships.

Hypertension is a long‐standing disease that often unknowingly leads to mortality of the patient. It has been demonstrated that integrated care model has a positive influence on systolic and diastolic blood pressure in hypertensive patients in a recent systematic review and meta‐analysis.^37^ The findings confirmed that group‐specific and disease‐specific models are better at improving hypertension and diabetes mellitus in contrast to individual care models. Zhao et al.^37^ also revealed that individual, group‐specific, and disease‐specific integrated care models better patients' glycated hemoglobin (HbA1C) in diabetes mellitus. The findings of the meta‐analysis suggested that the use of integrated care model intervention for a year was associated substantial improvement in HbA1C in comparison with 6‐month intervention. Huang et al.^38^ also reported similar findings. They documented significant decline in HbA1C upon 1‐year intervention. Just like the diabetes mellitus itself, the management is also a lengthy process and therefore as the duration of integrated care model stretches so does the improvement in HbA1C.^37^

Besides clinical outcomes, integrated care models have also been proven to improve the outcomes primarily propelled by financial forces such as hospitalization and length of hospital stay.^32^ An umbrella review by Damery et al.^39^ also reported decreased number of hospital admissions along with reduced number of days in hospital for patients managed by integrated care models. Another systematic review by Baxter et al.^40^ investigated 167 research studies and concluded significantly improved quality of care, high patient satisfaction, and accessibility to healthcare services with use of integrated care models.

## 5 WHAT IS CHRONIC CARE MODEL AND ITS KEY ELEMENTS?

The Chronic Care Model offers the ideal evidence‐centered framework for coordinating and enhancing the delivery of care to patients with chronic disease and make sure healthy communication between an educated and activated (with reference to his/her disease and management) patient and a trained healthcare providers team. Even though a number of methods have been used to apply research‐based suggestions into routine clinical setting, the Chronic Care Model has proven to be the standard model with success in diverse healthcare settings, with prime emphasis on diabetes mellitus. The notion of Chronic Care Model is that the dynamic and ongoing interactions between an informed and vested patient and family members and a trained healthcare provider team could result in better clinical outcomes. An activated patient can be defined as a patient who is adequately educated, encouraged, skilled, and entrusted to ensure effective self‐care and appropriate decision‐making regarding their disease. Equally, a trained healthcare provider team can be defined as a cadre of experts who has necessary patient information, suitable decision support system, and infrastructure required for provision of top‐quality health care. The Chronic Care Model works as a conceptual roadmap for reforming of a care from conventional acute diseases to a meticulously organized care of people with chronic diseases like diabetes mellitus.^10^

The Chronic Care Model is divided into six key elements that demands consideration for delivery of optimal clinical outcomes: organization of healthcare delivery system, self‐management support, decision support, delivery system design, clinical information systems, and community resources and policies.

### 5.1 Organization of healthcare delivery system

It refers to the development of an organizational ecosystem that provides and promotes highest quality health care and pledge to implement the Chronic Care Model across the healthcare organization. It includes active support from clinicians as leaders to facilitate improvement at every hierarchy of clinical setting, encouraging improvement approaches for complete system transformation, reassuring transparent and efficient dealing with errors and issues to enhance care, and formulating policies and guidelines that drive coordinated care in and across various clinical settings.^41^

### 5.2 Self‐management support

It refers to enabling, facilitating, and supporting patients with the disease to become self‐sufficient to manage their health and well‐being. It includes encouraging and realizing patient their role in their own health, providing patient with self‐care support package which encompass examination, objective setting, decision‐making, and following up, and arranging internal and external platforms to offer continual self‐care support.^41^

### 5.3 Decision support

It refers to supporting patient care that liaise with emerging and up‐to‐date scientific evidence, healthcare guidelines, and patients' choices. It includes accessibility of clinicians to up‐to‐date research‐driven guidelines for chronic disease care, ongoing informative sessions and programs for clinicians to emphasize use of standard yardsticks, ingraining clinicians with the habit of utilizing research‐driven guidelines into routine clinical settings, timely distributing research‐driven guidelines with patients to improve and elicit their participation, and incorporating disease experts in the process.^41^

### 5.4 Delivery system design

It refers to coordination of healthcare delivery process, that is, ensuring effective, competent care, and facilitation self‐management support. It includes consistent and planned clinic visits to integrate patient targets to assist them in maintaining ideal health and well‐being and guide healthcare systems to effectively manage resources, use of clinical and decision‐making skills of number of healthcare personnel in a Chronic Care Model team with assigned responsibilities, prearranged interactions to facilitate research‐guided care, offering case management facilities for clinically intricate patients, making sure steady follow‐up, and offering culturally competent health care.^41^

### 5.5 Clinical information systems

It refers to ongoing monitoring of data and clinical outcomes in such a way that enables healthcare providers in making decision regarding efficient care delivery. It includes an electronic health management system which helps clinicians in identifying and closely monitoring patients with chronic disease, giving appropriate nudges for patients and team members, enabling personalized care planning, sharing information with patients and team members to for care coordination, and evaluating the performance of care team.^41^

### 5.6 Community resources and policies

It refers to development and utilization of community‐related resources and public health guidelines to encourage healthy regimes and sustain health and well‐being. It includes motivating patients to take part in community outreach programs, establishing creative and problem‐solving partnerships with organizations in the community to facilitate and develop management interventions that satisfy required services, and promoting health policies that serve to better patient care.^41^

## 6 WHAT ARE THE EVIDENCE OF EFFECTIVENESS OF CHRONIC CARE MODEL FOR ADULTS WITH TYPE 2 DIABETES MELLITUS IN PRIMARY CARE ON IMPROVING PATIENT OUTCOMES?

The Chronic Care Model is an integrated care model that has proven benefits in improving the quality of diabetes mellitus care via implementation of its aforementioned six fundamental aspects.^42^ A 5‐year prospective cohort research study used a Risk Assessment and Management Program‐Diabetes Mellitus to manage patients with type 2 diabetes mellitus. They integrated components of Chronic Care Model of care planning based on risk stratification, multidisciplinary care support, preplanned surveillance of clinical complications, self‐care training, and smoking cessation. The study demonstrated substantial significant decrease in HbA1C, low‐density lipoprotein cholesterol, systolic blood pressure, diastolic blood pressure, and body mass index in intervention cohort. The risk of cardiovascular event, microvascular complications, and mortality reduced by 56.6%, 11.9%, and 66.1%, respectively.^15^ A randomized controlled trial by Clark et al.^43^ used an all‐encompassing diabetes program that involved risk stratum, action plan, planned follow‐ups, and patient participation in self‐management. Diabetes mellitus patients showed decline in HbA1C and blood pressure and high satisfaction with the integrated care program in primary care setting. In another study, the Risk Assessment and Management Program‐Diabetes Mellitus program was also noted to be as a cost‐effective intervention in the management of diabetes mellitus.^16^

### 6.1 HbA1C

The HbA1C is a clinical yardstick for close surveillance and management of patients with type 2 diabetes, with key objective of averting or delaying clinical complications like diabetic neuropathy, nephropathy, and retinopathy.^44^ A study by Piatt et al. (2006) divided subjects into three cohorts, that is, with Chronic Care Model intervention, other cohort with merely healthcare provider education, and the final cohort with usual care. At 12‐month postintervention, reduction in HbA1C was noted in the Chronic Care Model cohort compared with that of other cohorts. This outcome was also evident in patients who were actively self‐monitoring blood glucose. At 3‐year follow up, maintenance of HbA1C, that were observed at 12 months, was seen in the Chronic Care Model intervention cohort.^45^ A quantitative analysis from Pakistan in primary care of rural areas also showed mean difference in HbA1C of 0.83 after 6‐month intervention of Chronic Care Model in type 2 diabetes mellitus patients. The study used two components of the Chronic Care Model, that is, self‐management support and delivery system design.^46^ A number of systematic review and meta‐analyses of research studies present the evidence of effectiveness of Chronic Care Model in improving the clinical outcomes of these patients. They reported mean difference in HbA1C between intervention and routine care cohort of −0.07% to −0.5%.^41^, ^47^, ^48^, ^49^

### 6.2 Blood pressure

Hypertension is often existent with uncontrolled blood glucose in patients with type 2 diabetes mellitus.^50^ In a study by Hiss et al. (2007), there were two groups, one with intervention of personalized counseling, care plan, and case management for 6 months while other with usual care. Considerable improvement in mean systolic blood pressure was observed for former group. Interestingly, diastolic blood pressure improvement was only noted for patients who had >2 interactions with the team nurse.^51^ A recent systematic review and meta‐analysis by Goh et al. (2022) also confirmed more improvement in systolic and diastolic blood pressures in patients who received Chronic Care Model intervention. This can be attributed to self‐care and self‐management ability of patients to check their blood pressure.^41^ A randomized study in community health center in China also reported decline in diastolic blood pressure after 9 months upon Chronic Care Model intervention. The decline in diastolic blood pressure was from 75.06 ± 7.21 to 73.20 ± 5.94 mmHg.^5^

### 6.3 Low‐density lipoprotein cholesterol

Obesity is a key risk factor of development of type 2 diabetes mellitus.^52^ While a number of research studies including systematic reviews and meta‐analyses were unable to confirm the impact of Chronic Care Model intervention on low‐density lipoprotein cholesterol,^5^, ^41^, ^53^ only study that reported significant decrease in low‐density lipoprotein cholesterol postintervention was performed by White 2020.^54^

### 6.4 Body mass index

High body mass index is consistent with risk of diagnosis of type 2 diabetes mellitus.^55^ Ansari et al.^46^ reported mean difference in body mass index of 4.97 kg/m^2^ BMI after 6 months of Chronic Care Model intervention in type 2 diabetes mellitus patients in rural primary care setting of Pakistan. A study from China did not find any significant difference in mean body mass index for Chronic Care Model intervention and control cohort; however, significant decrease in waist circumference from 83.14 cm to 79.66 cm was documented.^5^ Carter et al.^56^ reported significant link between Chronic Care Model intervention and attaining a healthy body mass index.

### 6.5 Mortality and cost saving

Uncontrolled type 2 diabetes mellitus significantly increases the risk of all‐cause mortality.^57^ Only a handful of studies have studied the effectiveness of Chronic Care Model intervention in reducing the mortality and financial burden in patients with type 2 diabetes mellitus. A 5‐year Chronic Care Model effectiveness study on 53,436 patients in primary care with type 2 diabetes confirmed substantial reduction in mortality by 66.1%.^15^ Furthermore, similar research group reported reduced need to utilize healthcare services among type 2 diabetes mellitus patients in Chronic Care Model cohort, which ultimately saved US Dollar 7294 per patient during the study period.^16^

## 7 EMBRACING CHRONIC CARE MODEL IN PRIMARY CARE FOR PATIENTS WITH TYPE 2 DIABETES MELLITUS

Research evidence establishes that the integration of numerous elements of the Chronic Care Model had synergistic effect on outcomes of patients with type 2 diabetes mellitus than conventional single intervention methods.^11^, ^58^ The American Diabetes Association (ADA) Standards of Medical Care in Diabetes 2022^59^ also emphasizes that the scheme to managing diabetes mellitus in primary care setting must be in coherence with the Chronic Care Model, which focuses on patient‐centered care, integrated long duration therapeutic management, and continual collaborative interaction and goal setting between patient and Chronic Care Model team.

In days and years ahead, it is advised that from clinicians to nurses and every healthcare professional that are involved in any aspect of diabetes mellitus care must essentially identify barriers that inhibit our diabetes care clinical practice from being effective. By identifying the clinical needs of the diabetes mellitus patients in the primary care setting, we can significantly improve the communication and thereby chronic disease management. Ideal diabetes mellitus care relies on integrated care models like Chronic Care Model interventions that not only work to alert and empower diabetes mellitus care team but also patient themselves to take accountability of their health and well‐being in reaching desired health outcomes efficiently. Type 2 diabetes mellitus has been the prime concern, both in primary care setting and research, of Chronic Care Model owing to high cost and number of cases reaching pandemic dimensions. Driven by the type 2 diabetes mellitus itself, its complications, and resultant financial strain, implementation of the Chronic Care Model is a must in primary care settings and should be considered as a proxy diabetes healthcare delivery model.

## 8 CONCLUSION

In conclusion, the prevailing and burgeoning number of type 2 diabetes mellitus cases in years to come demands a systemized approach to tackle the challenges of diabetes mellitus management and adverse outcomes associated with this metabolic disorder. In this regard, the Chronic Care Model has been shown to be effective in improving the quality of care and outcomes of patients with type 2 diabetes mellitus. The Chronic Care Model is a well‐established framework that offers high‐quality care to patients with chronic diseases and improve clinical outcomes by emphasizing patient‐centered care, organized long‐lasting therapeutic management approaches to chronic disease and comorbidities, and constant cooperative communication and target setting between the patient and healthcare team. It focuses on acknowledging the patient needs, constructing an ecosystem for their care, and utilizing clinical information system‐guided processes to offer optimal diabetes mellitus care that will eventually congregate to better the quality of life of those afflicted. Propelled by the requirement to contain poor prognosis and healthcare expenditure prompted by type 2 diabetes mellitus; it is highly encouraged to implement the Chronic Care Model in primary care as a prototype of diabetes mellitus care delivery.

## AUTHOR CONTRIBUTIONS

AAA was involved in conceptualization, literature review, writing original draft, and reviewing and editing the final draft.

## CONFLICT OF INTEREST STATEMENT

The authors have stated explicitly that there are no conflicts of interest in connection with this article.

## ETHICS APPROVAL STATEMENT

None.

## PATIENT CONSENT STATEMENT

None.

## CLINICAL TRIAL REGISTRATION

None.
